# Long-Range Epistasis Mediated by Structural Change in a Model of Ligand Binding Proteins

**DOI:** 10.1371/journal.pone.0166739

**Published:** 2016-11-21

**Authors:** Erik D. Nelson, Nick V. Grishin

**Affiliations:** Howard Hughes Medical Institute, University of Texas Southwestern Medical Center, Room ND10.124, Dallas, Texas, United States of America; Weizmann Institute of Science, ISRAEL

## Abstract

Recent analyses of amino acid mutations in proteins reveal that mutations at many pairs of sites are epistatic—i.e., their effects on fitness are non—additive—the combined effect of two mutations being significantly larger or smaller than the sum of their effects considered independently. Interestingly, epistatic sites are not necessarily near each other in the folded structure of a protein, and may even be located on opposite sides of a molecule. However, the mechanistic reasons for long–range epistasis remain obscure. Here, we study long–range epistasis in proteins using a previously developed model in which off–lattice polymers are evolved under ligand binding constraints. Epistatic effects in the model are qualitatively similar to those recently reported for small proteins, and many are long–range. We find that a major reason for long–range epistasis is conformational change—a recurrent theme in both positive and negative epistasis being the transfer, or exchange of material between the ordered nucleus, which supports the binding site, and the liquid–like surface of a folded molecule. These local transitions in phase and folded structure are largely responsible for long–range epistasis in our model.

## Introduction

Epistasis in protein biophysics refers to the non–additive effects of amino acid mutations on protein folding and function [1]. An epistatic interaction is said to occur between two mutations when their combined effect on a trait is either larger or smaller than the sum of their effects considered independently. For example, a mutation that, by itself, damages the biochemical function of a protein may have a neutral or beneficial effect when considered in the presence of another mutation—a form of positive epistasis. Conversely, mutations that have a neutral, or nearly neutral effect on function individually, may produce a damaging effect in combination—a form of negative epistasis. Recent analyses of amino acid mutations in proteins reveal that mutations at many pairs of sites are epistatic, and suggest that epistasis plays a significant role in protein evolution [1–13]. Interestingly, epistatic sites are not necessarily in contact with each other in the folded state of a protein, and may even be located on opposite sides of a molecule. However, because the partially folded and mis–folded ensembles resulting from epistatic mutations are difficult to study, the mechanistic reasons for long–range epistasis remain somewhat obscure.

In this work, we investigate long–range epistasis using a previously developed model in which polymers were evolved to imitate the behavior of small ligand binding proteins [14]. The model re–capitulates basic properties of evolved proteins, such as folding to an ordered, soluble native structure, maintenance of amino acid sequence complexity [15], linear rates of amino acid change as a function of solvent exposure [16], packing density [17], and distance from the binding site [18], and linear rates of structure divergence as a function of the number of accepted mutations [19]. Below, we sample epistatic effects in evolved polymers by random selection of pair mutations, and we study the folded and mis–folded ensembles of single and double mutants in instances of significant epistasis—epistasis being measured in terms of the probability of folding a structure in which the binding site is correctly formed. Epistatic effects in the model resemble those reported by Olson et. al [2] for the small IgG–binding domain of protein G (protein GB1), and many are long–range. We find that a major reason for long–range epistasis is conformational change: In positive epistasis, either or both mutations disrupt the binding site, however, the double mutant folds to a locally re–configured native state in which the binding site is maintained. In negative epistasis, a single mutation leads to a neutral, or slightly deleterious change in native structure; This change conflicts with a second (formerly neutral, or slightly deleterious) mutation, leading to more frequent mis–folding of the binding site. A recurrent theme in both positive and negative epistasis is the transfer, or exchange of material between the ordered nucleus, which supports the binding site, and the disordered surface of a molecule, reminiscent of the theory of allostery in protein domains [20]. These local transitions in phase and structure are largely responsible for long–range epistasis in our model. Alternatively, neutral, or slightly deleterious mutations that preserve the native structure can conspire to frustrate formation of the binding site during folding. In this case, a mutation that increases the energy of the native fold relative to mis–folded states, or provides for greater conformational freedom during folding, amplifies the negative effect of a second mutation, presumably as a result of proximity to a thermodynamic phase transition [1, 2]. We find, however, that this process does not necessarily result in a complete change of phase.

In the following, we describe the distribution of epistatic effects in our model, and then explore the mechanisms of positive and negative epistasis in specific examples. First, we briefly outline our model; A more detailed description can be found in the Methods section.

## Model

The polymer model is a chain of point monomers that interact as low resolution amino acids via spherically symmetric pair potentials. Polymers evolve kinetically by Langevin dynamics. The fitness of a sequence is determined by folding N=127 polymer replicas on a parallel computer and analyzing the resulting ensemble of structures: Each replica is initiated from a random coil state below the folding temperature of a typical viable sequence. The amount of time allowed for folding is determined by the number of amino acids in a sequence, *N*, according to an estimate provided by Lin and Zewail [21]. The temperature is then reduced substantially, the replicas are equilibrated for a short period, and a final ensemble of folded and quenched structures, Γ, is recovered.

To obtain the sequences studied in this work, polymers were first evolved to recover an ordered (but otherwise un–restricted) folding domain, as determined by the Lindemann melting criterion [22]. To apply the Lindemann criterion, we select a structure $x^{⋆}$ and an ensemble $\Delta \Gamma^{⋆}$ of 3N/4 replicas to represent the dominant energy basin recovered by the folding procedure. Here, $x^{⋆}$ plays a role analogous to the equilibrium (lattice) positions of atoms in a crystal. The structure $x^{⋆}$ and the ensemble $\Delta \Gamma^{⋆}$ are selected to minimize the structurally aligned distance between $x^{⋆}$ and replicas **x**^*μ*^ included in $\Delta \Gamma^{⋆}$. To determine distance, the structures **x**^*μ*^ are aligned to $x^{⋆}$ by rotation, translation, and reflection through the closest 2*N*/3 pairs of monomers using methods described in reference [14]. Finally, sequences are selected to recover at least 15 ordered monomers, where order is measured by fluctuations of monomer positions $x_{j}^{\mu }$ in structures $x^{\mu }\in \Delta \Gamma^{⋆}$ against their positions $x_{j}^{⋆}$ in the reference structure $x^{⋆}$.

Under this condition, polymers spontaneously evolve ordered surface cavities (putative binding sites), resembling the active sites of small enzymes [23]. These “enzyme–like” sequences are subsequently evolved to recover an ordered binding site compatible with a model ligand, with the requirement of folding an ordered domain relaxed. To enforce the binding condition, the ligand is optimally docked onto the binding sites of replicas folded by a given sequence (see Methods). If the distances between amino acids in the binding site of a replica (including the ligand) are each within 1 Angstrom of their corresponding distances in a pre–defined target state, the replica is considered “active”. The fitness of a sequence is then defined by the fraction of active replicas, $P=N^{⋆}/N$, recovered by the folding and docking procedures.

In earlier work, we found that ligand binding, as defined above is, by itself, sufficient to maintain an ordered folding domain during evolution [14]: In order to maintain a given binding site structure against thermal fluctuations, it is necessary to maintain an ordered nucleus as a scaffolding to support the binding site. As a result, both binding affinity and thermodynamic stability are implicated in the fitness parameter P.

## Results

**Fig 1:**
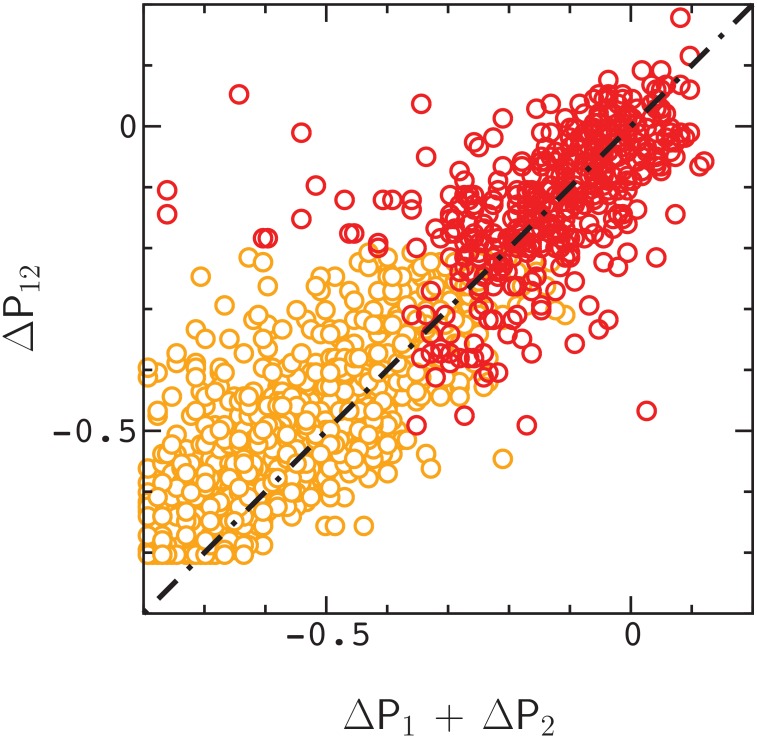
Correlation between ΔP_12_ and ΔP_1_ + ΔP_1_ for $≃1.5\times 10^{3}$ pair mutations randomly sampled from an evolved sequence folding to the ensemble shown in S1 Fig. The dashed line is a plot of the equation *ϵ* = 0. Data points that satisfy either ΔP_12_ ≥ λ, or ΔP_1_ ≥ λ and ΔP_2_ ≥ λ, with λ = −0.2 are colored red (see Text). The remaining data points are colored orange.

Below, we select two evolved sequences from these simulations to explore epistasis in the model (S1 and S2 Figs). In each sequence, pairs of sites are selected at random and subjected to random mutations roughly consistent with the genetic code (see S3 Fig and ref. [24]). For each pair of mutations, we compute the change in fitness, ΔP_*ν*_ = P_*ν*_ − P_0_ where P_*ν*_ is the fitness of a (single or double) mutant sequence, and P_0_ is the fitness of the initial evolved sequence. Epistasis is measured as

$$\epsilon \;=\;\Delta P_{12}\;-\;\Delta P_{1}\;-\;\Delta P_{2}$$ (1)

in obvious notation. Fig 1 provides a correlation plot of ΔP_12_ versus ΔP_1_ + ΔP_2_ for the initial sequence in S1 Fig. The dashed line is a plot of the equation *ϵ* = 0.

**Fig 2:**
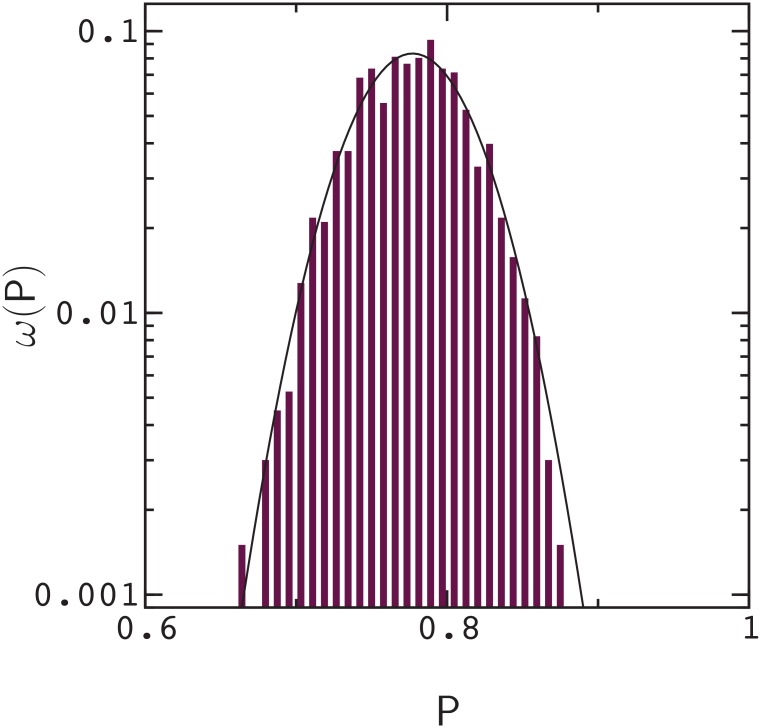
Distribution of fitness values, *ω*(P), obtained by the folding and docking procedure for an evolved sequence. The distribution is based on 10^3^ measurements. The solid line is a fit to a Gaussian distribution with width σ(P)≃0.037. Similar results are obtained for mutated sequences with lower mean fitness.

To estimate the uncertainty, or error in a single fitness measurement (i.e., against the value that would be obtained in the limit N→∞), we computed the width, *σ*(P), of the fitness distribution, *ω*(P), for a number of different sequences. The distribution of fitness values for an evolved sequence is shown in Fig 2. From these considerations, we find that the typical error, *δ*P, in a measurement of P (i.e., the typical width of a distribution, 〈 *σ*(P)〉) is about *δ*P ≃ 0.037. The value of P_0_ used in Fig 1 is based on 10^3^ measurements, and consequently, the errors in ΔP_*ν*_ are essentially the same as the errors in P_*ν*_. If errors in P_*ν*_ are considered independent, the error in ΔP_1_ + ΔP_2_ can be estimated as $\sqrt{2}\;\delta P$, and the error in *ϵ* can be estimated as $\delta \epsilon ≃\sqrt{3}\;\delta P$.

**Fig 3:**
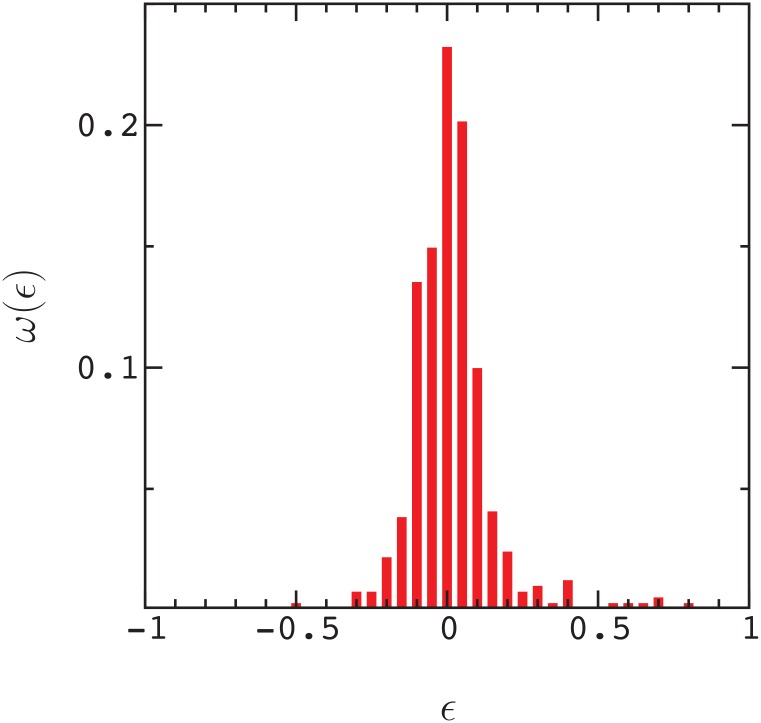
Distribution of epistasis vales, *ω*(*ϵ*), for (red) data points satisfying the condition defined in Fig 1. The width of the distribution is σ(ϵ)≃0.14.

**Fig 4:**
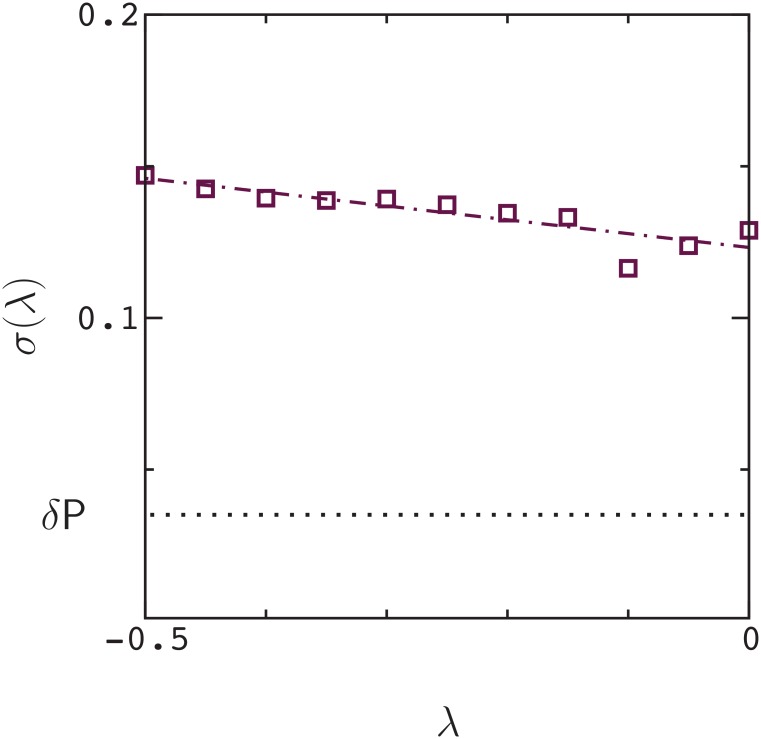
Width *σ*(*ϵ*) of the distribution *ω*(*ϵ*) as a function of λ.

In Fig 3, we plot the distribution of *ϵ* values, *ω*(*ϵ*), for points in Fig 1 that satisfy either ΔP_12_ ≥ λ, or ΔP_1_ ≥ λ and ΔP_2_ ≥ λ, with λ = −0.2 (i.e., for positive epistasis, the double mutant is neutral, or slightly deleterious, and likewise for single mutants in negative epistasis. This cut through the distribution is somewhat arbitrary, and is simply intended to include mutants that have a chance of becoming fixed in evolution). The width of the distribution, *σ*(*ϵ*), is about 3 times the error in a measurement of *ϵ*. This result is maintained for essentially all values of λ (Fig 4). As a result, about 30 percent of the data points exhibit statistically significant epistasis ($|\epsilon |\tilde{>}3\delta \epsilon$) in rough agreement with the results of Olson et. al [2] for protein GB1 (see also ref. [1]).

**Fig 5:**
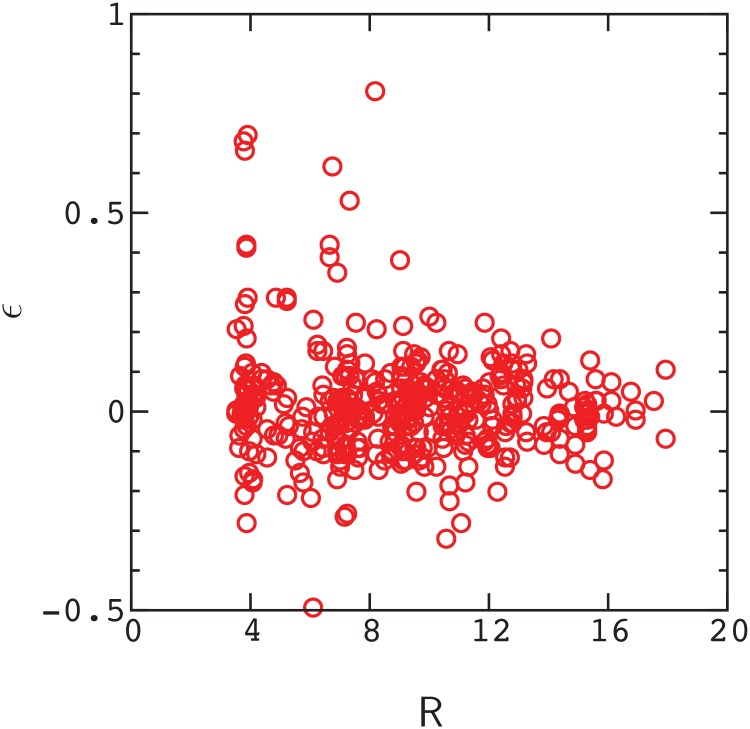
Distribution of epistasis values as a function of the distance, R, between mutations in the reference fold in S1 Fig. Data points satisfy the condition defined in Fig 1. Peaks in the density of points at R∼l, etc. reflect typical distances between pairs of monomers in the reference fold.

Finally, in Fig 5 we plot the distribution of *ϵ* values for points in Fig 1 as a function of the distance, R, between mutations in the initial fold. Amino acids begin to interact directly when R < 1.5*l*, where *l* = 3.8 Angstroms is the length of a polymer bond (see Methods). As is evident by inspection of Fig 5, many of the samples exhibit pronounced long–range epistasis, with R > 1.5*l* and *ϵ* ≫ *δϵ*. The variation in *ϵ* values decreases with R, similar to the results for protein GB1, however, positive epistasis is more prevalent than negative epistasis in the model. Similar results to those above are obtained for the sequence in S2 Fig.

To conclude our analysis, we examined the folded ensembles of 12 samples exhibiting various levels of positive and negative epistasis. For each sample, fitness values, P_*ν*_, were re–computed by averaging 10^2^ measurements to minimize the error in ΔP_*ν*_. Samples were selected essentially at random to include a range of values of *ϵ*.

**Fig 6:**
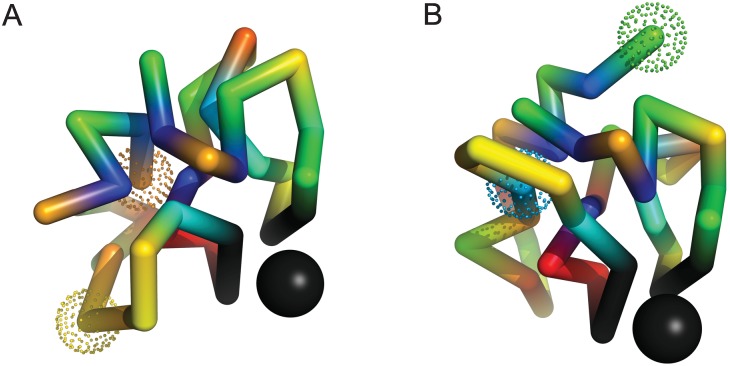
An example of long–range positive epistasis with *ϵ* = 0.77. Panel (A) describes the reference fold obtained for the initial sequence; Panel (B) describes the reference fold obtained for the double mutant. Dotted spheres indicate the positions of mutated amino acids. Individual mutations, R1 (orange) → G1 (green) and T12 (yellow) → I12 (light blue) are strongly deleterious, with $\Delta P_{1}≃-0.61$ and $\Delta P_{2}≃-0.29$ respectively ($\Delta P_{12}≃-0.13$). R1 is ordered (T12 is disordered) in the initial ensemble, and I12 is ordered (G1 is disordered) in the double mutant ensemble. The distance between mutated positions in panel (A) is R≃8 Angstroms. Folded ensembles for the initial and mutated sequences are shown in S4 Fig.

In all 6 instances of positive epistasis (in particular, even when epistasis is weak) the double mutant is found to adopt a locally re–configured native structure that preserves the binding complex. Fig 6 shows a typical example of long–range positive epistasis; Panels in this figure describe (A) the folded structure of the initial, un–mutated sequence, and (B) the folded structure of the double mutant (mutated sites are indicated by dotted spheres). Amino acids (monomers) are colored blue, light blue, blue–green, green, yellow, orange, and red, in order of increasing affinity to solvent. Binding site monomers are colored black. Charged amino acids R (orange), K, D and E (red) interact strongly, and play a similar role to hydrophobic amino acid types such as W, V, L, and I (blue) in stabilizing the binding sites of evolved sequences. To aid interpretation of Fig 6, the folded ensembles of the initial and mutated sequences are provided in S4 Fig. This scheme is repeated for the remaining examples studied below.

In this example, both single mutants recover disordered ensembles in which the binding site is disrupted: The mutation R1 → G1 destabilizes the charged part of the ordered scaffolding (red and orange) that supports the binding site, removing a charged amino acid; The mutation T12 → I12, while adding a hydrophobic amino acid, leads to competing nuclear structures. In the double mutant, the destabilizing effect of the first mutation is compensated by the second through local changes in the structure of chain segments containing the mutated sites, leading to an exchange of material between the ordered nucleus and less ordered (surface) regions of the folded ensembles (S4 Fig).

**Fig 7:**
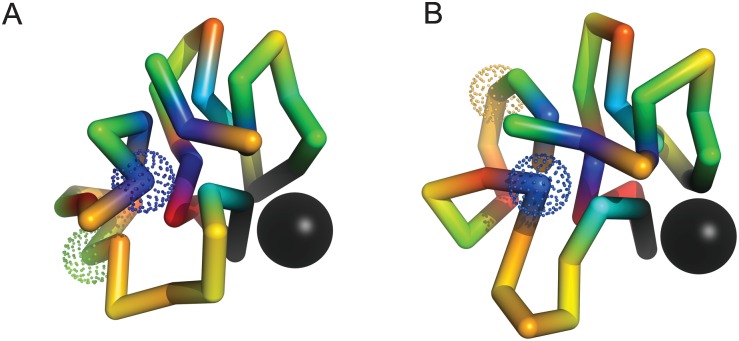
An example of long–range positive epistasis with *ϵ* = 0.25. Individual mutations, W4 (blue) → S4 (yellow) and G10 (green) → V10 (blue) are slightly deleterious, with $\Delta P_{1}≃-0.17$ and $\Delta P_{2}≃-0.11$ respectively ($\Delta P_{12}≃-0.04$). W4 is ordered (G10 is disordered) in the initial ensemble, and V10 is ordered (S4 is disordered) in the double mutant ensemble. In the double mutant, V10 occupies the nuclear site originally occupied by W4. The distance between mutated positions in panel (A) is R≃9 Angstroms. Folded ensembles for the initial and mutated sequences are shown in S5 Fig.

**Fig 8:**
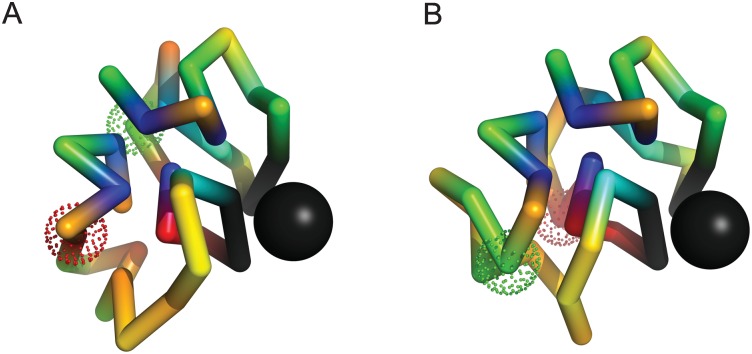
An example of long–range positive epistasis with ϵ≃0.2. Individual mutations E6 (red) → G6 (green) and A23 (green) → E23 (red) are neutral and deleterious, with $\Delta P_{1}≃0$ and $\Delta P_{2}≃-0.24$ respectively ($\Delta P_{12}≃-0.04$). E6 and A23 are ordered in the initial ensemble, while E23 is ordered and G6 is disordered in the double mutant ensemble. In the double mutant, material is transferred from ordered to disordered phase regions of the ensembles by the mutation E6 → G6, while the mutation A23 → E23 enhances the stability of the nuclear scaffolding. The distance between mutated positions in panel (A) is R≃8 Angstroms. Folded ensembles for the initial and mutated sequences are shown in S6 Fig.

A similar example is shown in Fig 7. Here, the mutation W4 → S4 removes a hydrophobic monomer from the nuclear scaffolding leading to large fluctuations in the binding site; The mutation G10 → V10, while adding a hydrophobic amino acid to the nucleus, leads to competing nuclear structures during folding (originally, G10 is disordered). In the double mutant, V10 replaces W4 in the nucleus, and the mutated amino acid S4 is disordered. As in Fig 6, the double mutation leads to the re–configuration of loops containing the mutated sites, exchanging material between ordered and disordered phase regions of the folded ensembles. A slightly different mechanism of long–range positive epistasis is shown in Fig 8. Here, the mutation A23 → E23 has a stabilizing effect once the charged amino acid E6 is removed from the nuclear scaffolding by the mutation E6 → G6. Both A23 and E23 are ordered in their respective ensembles. In the double mutant, material is transferred from ordered to disordered phase regions of the folded ensembles by the mutation E6 → G6.

**Fig 9:**
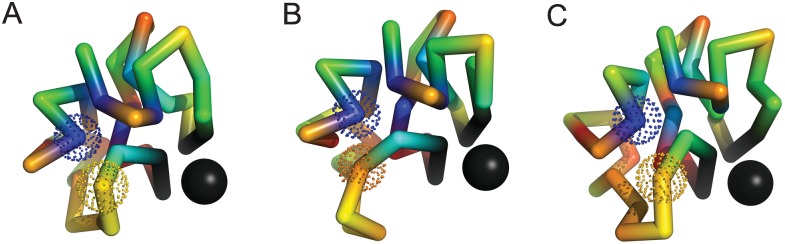
An example of long–range negative epistasis with ϵ≃−0.32. Panel (A) describes the reference fold obtained for the initial sequence, while panels (B) and (C) describe the reference folds obtained for the single mutants; Individual mutations T15 (yellow) → R15 (orange) and W4 (blue) → C4 (blue) in panels (B) and (C) are slightly deleterious and neutral, with $\Delta P_{1}≃-0.17$ and $\Delta P_{2}≃0.02$ respectively ($\Delta P_{12}≃-0.47$). W4 is ordered (T15 is disordered) in the initial ensemble, while R15 and C4 are ordered in the single mutant ensembles. The mutation T15 → R15 brings the mutant amino acid R15 in contact with W4. However, this structure (B) conflicts with the mutation W4 → C4 (the interaction between R and C is repulsive). The distance between mutated positions in panel (A) is R≃6 Angstroms. Folded ensembles for the initial and mutated sequences are shown in S7 Fig.

To study negative epistasis, we selected samples in which single mutations are neutral or slightly deleterious. Fig 9 provides an example of long–range negative epistasis in which the structural change caused by one mutation is frustrated by a second, formerly neutral mutation. Panel (A) describes the folded structure of the initial sequence, while panels (B) and (C) describe the folded structures of the single mutants T15 → R15 and W4 → C4 respectively. The mutation T15 → R15 adds a charged amino acid to the nuclear scaffolding, which brings the mutant amino acid R15 in contact with W4; The interaction between R15 and W4 is attractive, and the amino acid T15 is disordered in the initial ensemble. The second mutation, W4 → C4, is neutral, and leaves the native structure essentially intact. However, the interaction between C4 and R15 is repulsive, which frustrates the transfer of R15 to the nucleus in the double mutant, disrupting the binding site. This pattern is repeated in 2 of the examples we studied.

**Fig 10:**
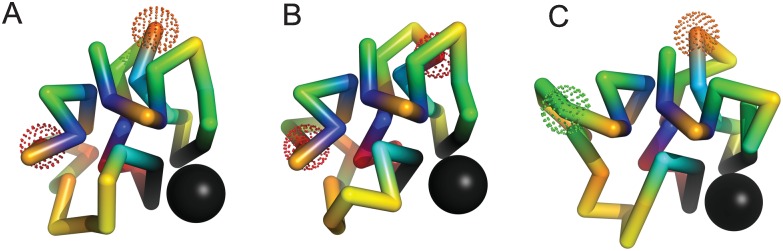
An example of weak but very long–range negative epistasis with ϵ≃−0.11. Individual mutations Q25 (orange) → E25 (red) and E6 (red) → G6 (green) in panels (B) and (C) are slightly deleterious and neutral, with $\Delta P_{1}≃-0.11$ and $\Delta P_{2}≃0$ respectively ($\Delta P_{12}≃-0.22$). E6 is ordered (Q25 is disordered) in the initial ensemble, and E25 is ordered (G6 is disordered) in the single mutant ensembles. In the double mutant, the removal of E6 allows E25 to form favorable contacts with other charged monomers during folding, which leads to more frequent mis–folding of the binding site, frustrating the exchange of material between ordered and disordered phases. The distance between mutated positions in panel (A) is R≃12.5 Angstroms. Folded ensembles for the initial and mutated sequences are shown in S8 Fig.

Fig 10 describes an example of weak, but very long–range negative epistasis in which an exchange of material between ordered and disordered phases is frustrated. Again, panel (A) describes the folded structure of the initial sequence, while panels (B) and (C) describe the folded structures of the single mutants Q25 → E25 and E6 → G6 respectively. The mutation E6 → G6 removes a charged amino acid from the nuclear scaffolding. In the mutant ensemble, the amino acid G6 is disordered, however, this has no effect on fitness. The mutation Q25 → E25 adds an ordered charged monomer; However, in the mutant ensemble, the interactions between E25 and its neighbors are almost all repulsive. In the double mutant, the removal of E6 allows E25 to form favorable contacts with other charged monomers during folding, which leads to more frequent mis–folding of the binding site.

The remaining samples of negative epistasis conform to the process described earlier above, in which a neutral, or slightly deleterious mutation that preserves the native structure amplifies the negative effect of another. An example of this effect exhibiting strong epistasis is provided in S9 Fig. In this sample, the mutation, D19 → Y19, reduces the specificity of interactions with its neighbors, allowing for greater conformational freedom of the binding site, making it more susceptible to the negative effect of the mutation, N13 → D13; The mutated amino acids Y19 and D13 form favorable contacts in mis–folded states of the replica ensemble.

## Discussion

To summarize, many instances of long–range epistasis in our study involve local structural changes that transfer or exchange material between ordered and disordered phase regions of the initial and double mutant ensembles. In positive epistasis, the loss of an amino acid on the ordered scaffolding which supports the binding site creates a “vacancy” that is compensated by a second amino acid in the double mutant. The second amino acid either occupies a site close to the vacancy (Fig 6) or fills the vacancy with a similar amino acid (Fig 7). In negative epistasis, the filling of an existing vacancy on the ordered scaffolding is frustrated by a second mutation (Fig 9).

In these examples, epistatic mutations connect local environments in a folded molecule that are dis–connected in the initial evolved structure. This may explain why long–range epistatic interactions are difficult to predict [25]: If the environments of mutated residues change significantly in epistasis, the information needed to predict epistatic interactions will depend on the environments of residues formed in the mutant structures, which will often be excluded from the evolutionary record by purifying selection. In addition, because computer modeling techniques are not sufficiently advanced to predict these changes, it is actually necessary to solve the folded structures of the mutant proteins to reveal the causes of epistasis. As a result, these effects, if they are detected, are very difficult to interpret, and may be more prevalent than expected.

Of course, our model is rather small, and neglects many of the constraints present in folded proteins, such as secondary structure. While this is not an obstacle for modeling short proteins (which are commonly devoid of significant secondary structure), secondary structure provides additional stiffness in larger proteins, which can lead to longer range collective effects such as allostery [26], and more exotic mechanisms for epistasis. Accordingly, the structural changes exhibited by our model are probably limited to more flexible regions in larger proteins.

## Methods

The polymer model is a chain of point monomers that interact as low resolution amino acids via spherically symmetric potentials. Interactions along the chain are described by potentials of the form,

$$U^{\kappa }\left(r\right)\;=\;\frac{\kappa }{2}\;{\left(r-l\right)}^{2}$$ (2)

where *r* is the distance between monomers, *l* is the equilibrium length of a link, and *κ* is a constant (see below).

Interactions between non–adjacent monomers along the chain are constructed from the unit Morse potential,

μ(r)=exp(-2α(r-l))-2exp(-α(r-l)) (3)

**Fig 11:**
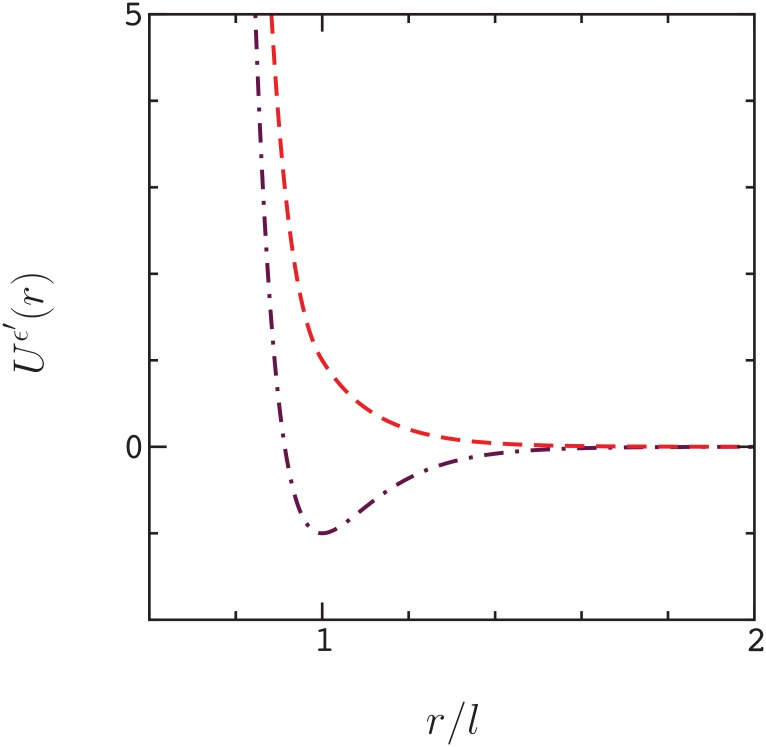
Potential functions, *U*^*ϵ*′^(*r*), for cross-chain interactions at unit core strength, *ϵ* = 1, unit attraction, *ϵ*′ = −1 (dot–dashed line) and unit repulsion, *ϵ*′ = 1 (dashed line).

The attractive minimum of the Morse potential occurs at *r* = *l*. Let

$$\mu^{r\leq l}\left(r\right)\;=\;\vartheta \left(l-r\right)\;\mu \left(r\right)$$ (4)

and

$$\mu^{r\geq l}\left(r\right)\;=\;\vartheta \left(r-l\right)\;\mu \left(r\right)$$ (5)

denote the components of the Morse potential in either side of the minimum, where *ϑ* is the unit step function. The potentials for attractive and repulsive amino acid interactions are constructed as,

$$U^{\epsilon^{\prime }\leq 0}\left(r\right)\;=\;\epsilon \;\mu^{r\leq l}\left(r\right)\;+\;\left(\epsilon \;+\;\epsilon^{\prime }\right)\;\vartheta \left(l-r\right)\;-\;\epsilon^{\prime }\;\mu^{r\geq l}\left(r\right)$$ (6)

and

$$U^{\epsilon^{\prime }\geq 0}\left(r\right)\;=\;\epsilon \;\mu^{r\leq l}\left(r\right)\;+\;\epsilon \;\vartheta \left(l-r\right)\;+\;\epsilon^{\prime }\;\exp\left(-\alpha \left(r-l\right)\right)$$ (7)

respectively (Fig 11).

Each potential consists of an excluded volume part, *ϵμ*^*r* ≤ *l*^(*r*) + *ϵϑ*(*l* − *r*), modulated by the parameter *ϵ*, and a sequence dependent part, modulated by the parameter *ϵ*′; The parameter *ϵ*′ takes on different values,

$$\epsilon^{\prime }\;=\;\epsilon \;E_{\mu \nu }/E_{o}$$ (8)

depending on the amino acid types involved in an interaction, where *E*_*μν*_ is the energy of a contact between amino acids *μ* and *ν* defined by the empirical parameters in reference [27], and *E*_o_ = 〈|*E_μν_*_≥*μ*_ |〉 is the average strength of an interaction (the empirical parameters are obtained by re–scaling the Miyazawa–Jernigan parameters [28] using threonine as a reference solvent [27]). The potentials for unit strength attractive and repulsive interactions are plotted in Fig 11.

To describe polymer kinetics, we integrate the Langevin equation using the method of van Gunsteren and Berendsen [29], with monomer mass *m* = 1.66⋅10^−22^ g, friction coefficient *γ* = 10 ps^−1^, and integration time step, Δ*t* = 0.01 ps. The parameters used to define the potentials are *l* = 3.8 Angstroms, *κ* = 11 *k*_*B*_ *T*_0_, *α* = 2.1 Angstroms^−1^, and *ϵ* = 2 *k*_*B*_ *T*_0_, where *k*_*B*_ is Boltzmann’s constant and *T*_0_ = 302.15 Kelvin.

Folding is initiated from a random coil state below the folding transition temperature of a typical evolved sequence, which we estimate as *T*_*f*_ ∼ 1.25*T*_0_ from specific heat data. The time allowed for folding is determined by the length of the polymer according to the estimate of Lin and Zewail [21],

$$t_{f}\;=\;N{\left(\frac{3}{e}\right)}^{N}\;\Delta t_{f}$$ (9)

where Δ*t*_*f*_ = 10 ps roughly describes the timescale for positional exchanges among monomers on the surfaces of polymer nuclei. Following this step, the replicas are equilibrated for a short time *t*_*q*_ = *t*_*f*_/3 at temperatures *T*_1_ = 218.2 Kelvin and *T*_2_ = 134.3 Kelvin.

To determine the number of active replicas in the folded ensemble, it is necessary to dock the model ligand onto the binding site structures recovered by replicas in the folding procedure. To accomplish this, the folded structure of a replica is enclosed in a spherical shell consisting of ∼10^4^ evenly distributed points [30]. We then measure, and record the energy of the target ligand (in this work, a single monomer) at each point on this shell. In this procedure, interactions with monomers in the binding site group are considered attractive, and are described by unit Morse potential, *μ*(*r*), while interactions with monomers not included in the binding site group are described by the repulsive core of the Morse potential, *μ*^*r* ≤ *l*^(*r*). The radius of the shell is reduced, and the energies are re–computed at each point, iteratively, until the shell lies inside the folded structure of the replica. The structure of the binding site complex (i.e. binding site plus ligand) is determined from this sweep as the configuration with minimal energy. A docked replica is considered active when the distances between amino acids in the binding complex are each within 1 Angstrom of their corresponding distances in a pre–defined target state, determined by averaging the states of replicas with properly formed binding sites recovered by a selected initial (evolved) sequence. The structures used to represent the dominant energy basin recovered by a sequence are obtained using the structural alignment procedure described in ref. [14].

## Supporting Information

S1 Fig(A) Folded structure $x^{⋆}$, and (B) sample of the folded ensemble $\Delta \Gamma^{⋆}$ recovered by a selected sequence evolved under ligand binding conditions.Amino acids (monomers) are colored blue, light blue, blue–green, green, yellow, orange, and red, in order of increasing affinity to solvent. The binding site monomers and the target ligand (here, a single monomer) are colored black. The ensemble $\Delta \Gamma^{⋆}$ is aligned to $x^{⋆}$ using methods described in ref. [14].(TIFF)

S2 Fig (A) Folded structure, and (B) sample of the folded ensemble recovered by a second evolved sequence. Panels of the figure are described as in S1 Fig.
(TIFF)

S3 Fig Amino acid exchange probabilities, *p*(*μ*, *ν*) = *A*_*μν*_ / ∑_*ν*_ *A*_*μν*_, for evolved sequences in ref. [14], where *A*_*μν*_ is the number of transitions recorded between amino acids *μ* and *ν*. Model values are indicated by filled red circles. Empirical values obtained from the data of Dayhoff et. al [24] are indicated by open blue circles. The value of *p*(*μ*, *ν*) is indicated by the radius of the corresponding circle. In random sampling of pair mutations, amino acid transitions are allowed when *p*(*μ*, *ν*)>0.
(TIFF)

S4 FigFolded ensembles of initial and mutated sequences corresponding to Fig 6.Panel (U) corresponds to the initial, un–mutated sequence. Panels (1) and (2) correspond to the single mutants R1 (orange) → G1 (green) and T12 (yellow) → I12 (light blue), respectively. Panel (12) corresponds to the double mutant. Dotted spheres indicate the positions of mutated amino acids. Each ensemble $\Delta \Gamma^{⋆}$ is aligned to its corresponding reference fold, $x^{⋆}$, using methods described in ref. [14] For clarity, each figure panel includes the 30 closest structures to $x^{⋆}$. Ensembles are rotated to reveal the positions of mutated monomers.(TIFF)

S5 Fig Folded ensembles of initial and mutated sequences corresponding to Fig 7. Panel (U) corresponds to the initial, un–mutated sequence. Panels (1) and (2) correspond to the single mutants W4 (blue) → S4 (yellow) and G10 (green) → V10 (blue), respectively. Panel (12) corresponds to the double mutant. Ensembles are arranged as described in S5 Fig.
(TIFF)

S6 Fig Folded ensembles of initial and mutated sequences corresponding to Fig 8. Panel (U) corresponds to the initial, un–mutated sequence. Panels (1) and (2) correspond to the single mutants E6 (red) → G6 (green) and A23 (green) → E23 (red), respectively. Panel (12) corresponds to the double mutant. Ensembles are arranged as described in S5 Fig.
(TIFF)

S7 Fig Folded ensembles of initial and mutated sequences corresponding to Fig 9. Panel (U) corresponds to the initial, un–mutated sequence. Panels (1) and (2) correspond to the single mutants T15 (yellow) → R15 (orange) and W4 (blue) → C4 (blue), respectively. Panel (12) corresponds to the double mutant. Ensembles are arranged as described in S5 Fig.
(TIFF)

S8 Fig Folded ensembles of initial and mutated sequences corresponding to Fig 10. Panel (U) corresponds to the initial, un–mutated sequence. Panels (1) and (2) correspond to the single mutants Q25 (orange) → E25 (red) and E6 (red) → G6 (green), respectively. Panel (12) corresponds to the double mutant. Ensembles are arranged as described in S5 Fig.
(TIFF)

S9 FigAn example of long–range negative epistasis with ϵ≃−0.26.Individual mutations N13 (yellow) → D13 (red) and D19 (red) → Y19 (blue–green) in panels (B) and (C) are nearly neutral, with $\Delta P_{1}≃-0.07$ and $\Delta P_{2}≃-0.1$ respectively ($\Delta P_{12}≃-0.43$). Both N13 and D19 are ordered in the initial ensemble, and both D13 and Y19 are ordered in the single mutant ensembles. Single mutations do not significantly alter the reference structure of the initial sequence. In the double mutant, the mutation, D19 → Y19, reduces the specificity of interactions with its neighbors, allowing for greater conformational freedom of the binding site, making it more susceptible to the negative effect of the mutation, N13 → D13; The mutated amino acids Y19 and D13 form favorable contacts during folding, which disrupts the binding site in the quenched ensemble. The distance between mutated positions in panel (A) is R≃7.3 Angstroms.(TIFF)

S1 File Text files of the data points and structures shown in Figs 1–10 and S1–S9 Figs. Structural ensembles are provided in Protein Data Bank (PDB) format. In each ensemble file, structures (models) are aligned to the reference fold (first model in a given file), and are arranged in order of decreasing alignment quality. Structures are aligned through the closest 2*N*/3 monomers as described in the Text.
(GZ) Click here for additional data file.
