# Supplementary figures and images for: Long-Range Epistasis Mediated by Structural Change in a Model of Ligand Binding Proteins
Source: PLoS One. 2016 Nov 21;11(11):e0166739. doi: 10.1371/journal.pone.0166739 (PMC5117711; doi:10.1371/journal.pone.0166739)

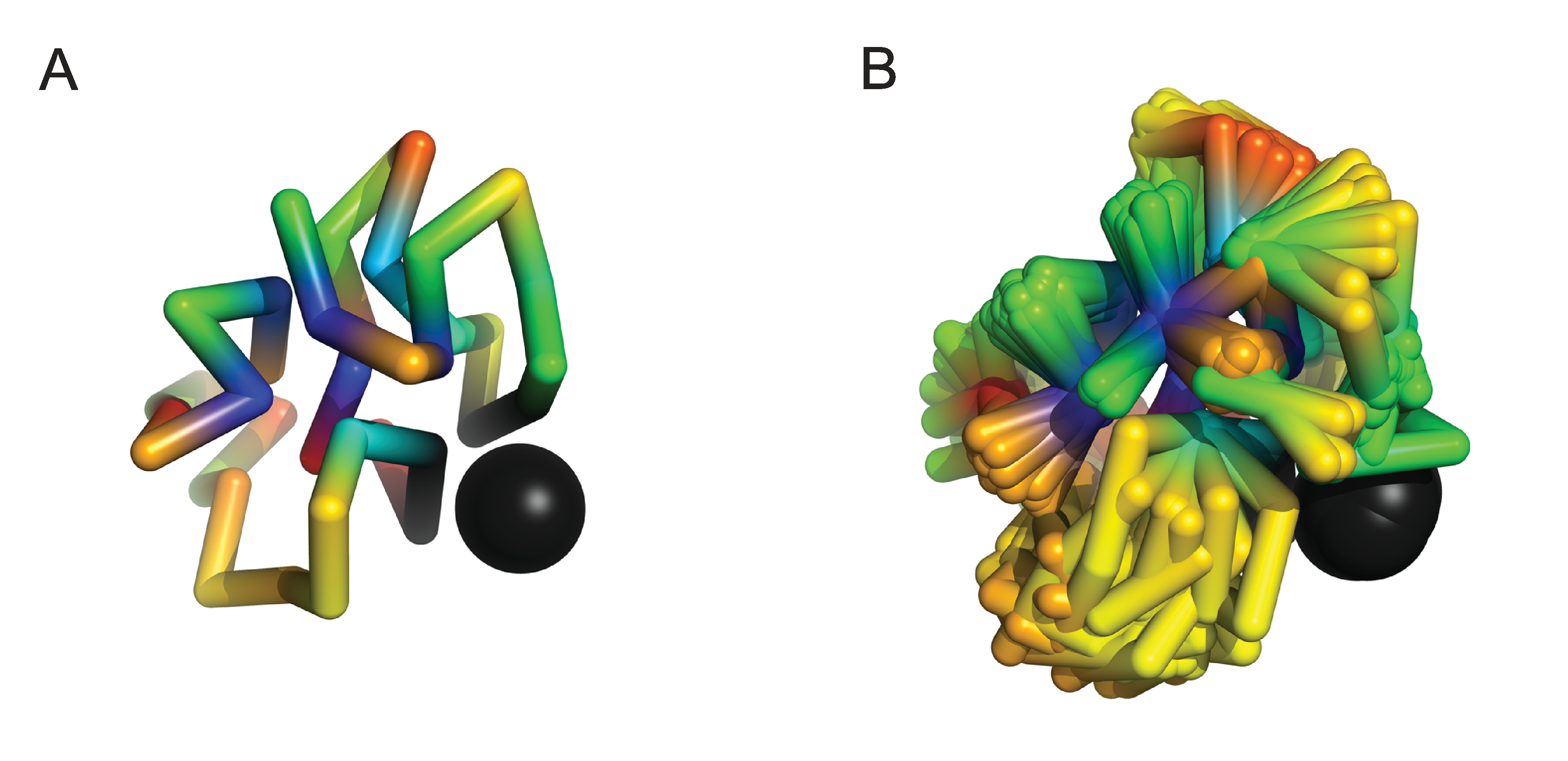

Supplement: S1 Fig — Amino acids (monomers) are colored blue, light blue, blue–green, green, yellow, orange, and red, in order of increasing affinity to solvent. The binding site monomers and the target ligand (here, a single monomer) are colored black. The ensemble ΔΓ⋆ is aligned to x⋆ using methods described in ref. [14]. (TIFF) [file pone.0166739.s001.tiff]

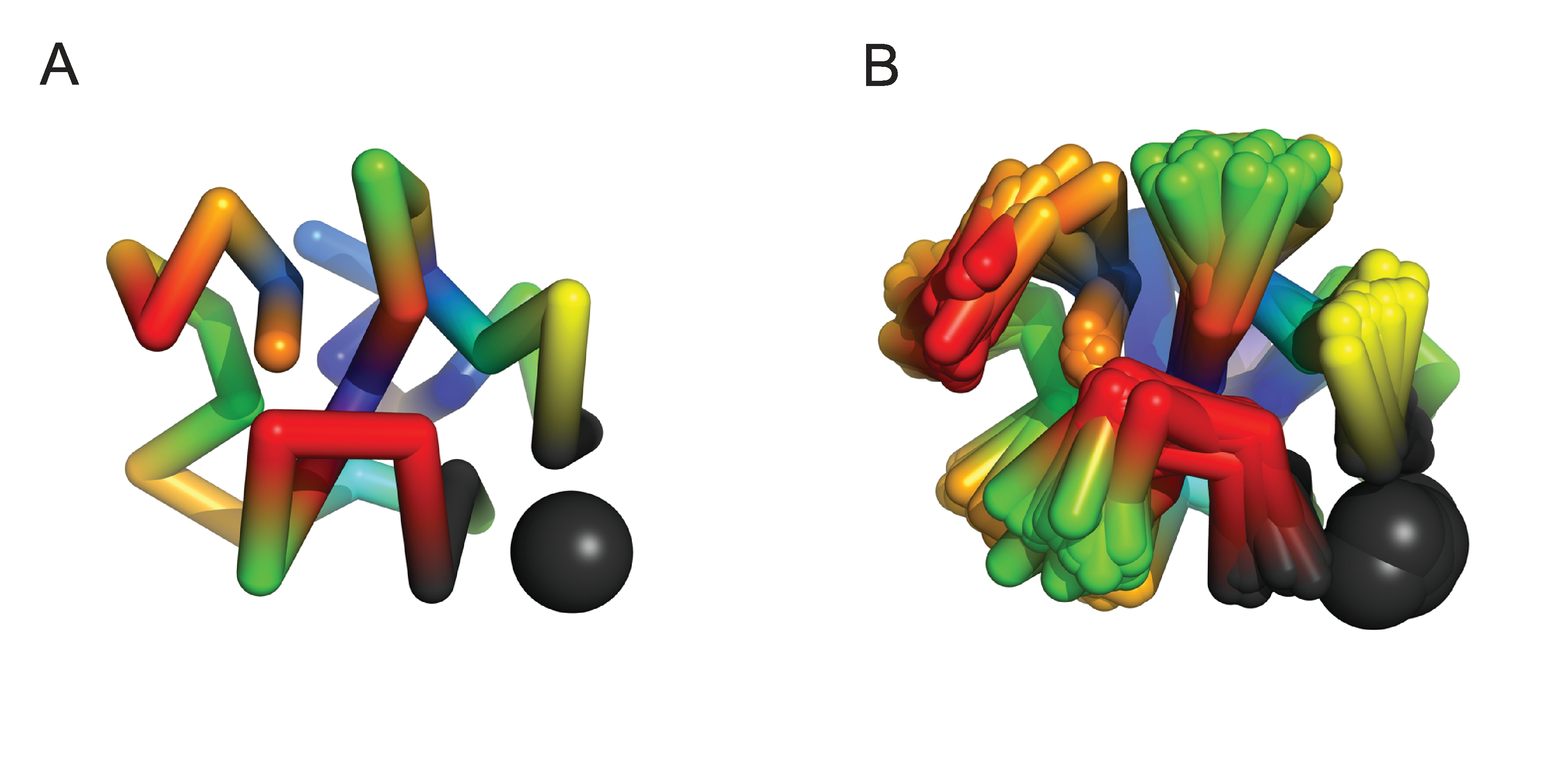

Supplement: S2 Fig — Panels of the figure are described as in S1 Fig. (TIFF) [file pone.0166739.s002.tiff]

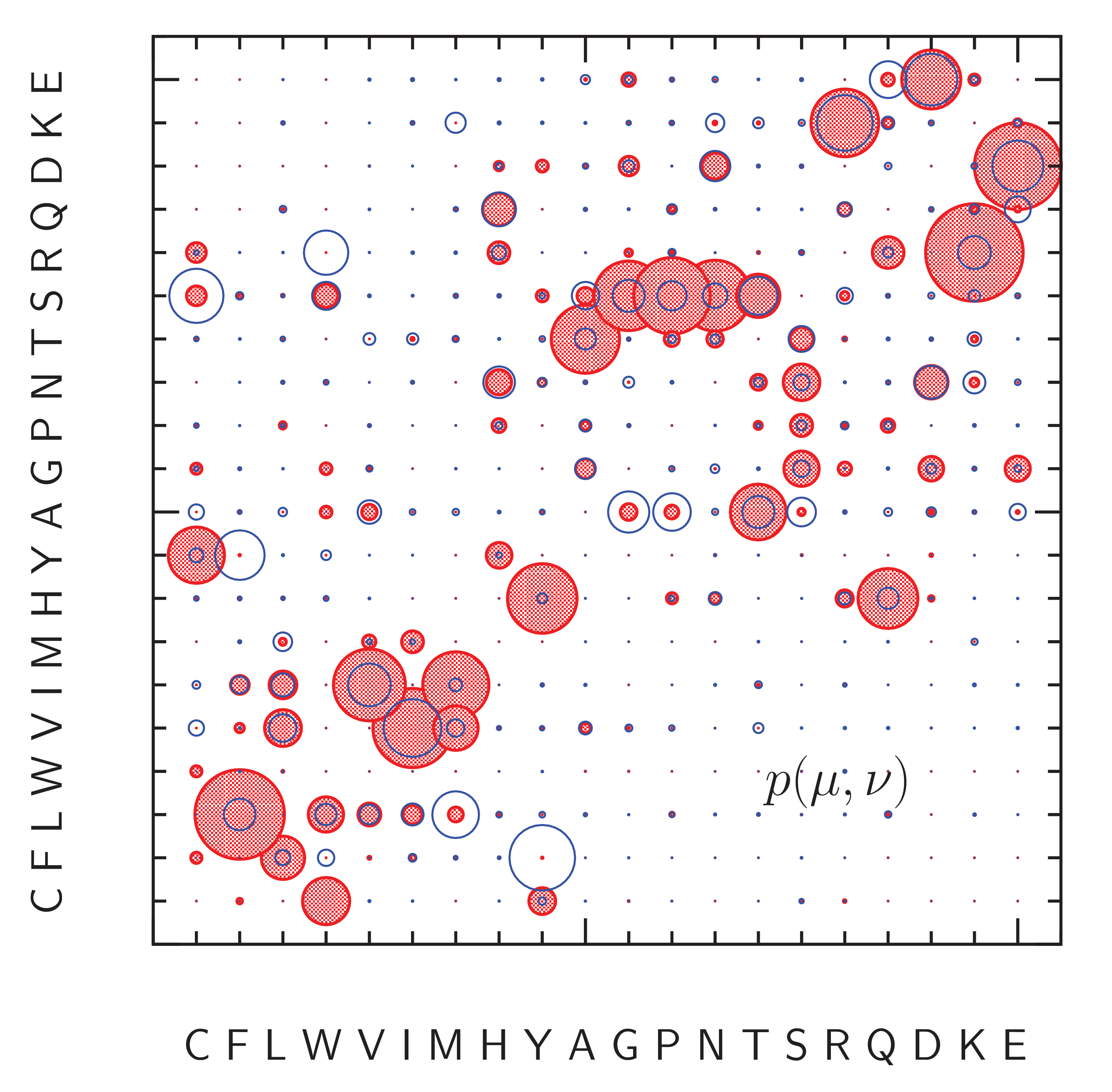

Supplement: S3 Fig — Model values are indicated by filled red circles. Empirical values obtained from the data of Dayhoff et. al [24] are indicated by open blue circles. The value of p(μ, ν) is indicated by the radius of the corresponding circle. In random sampling of pair mutations, amino acid transitions are allowed when p(μ, ν)>0. (TIFF) [file pone.0166739.s003.tiff]

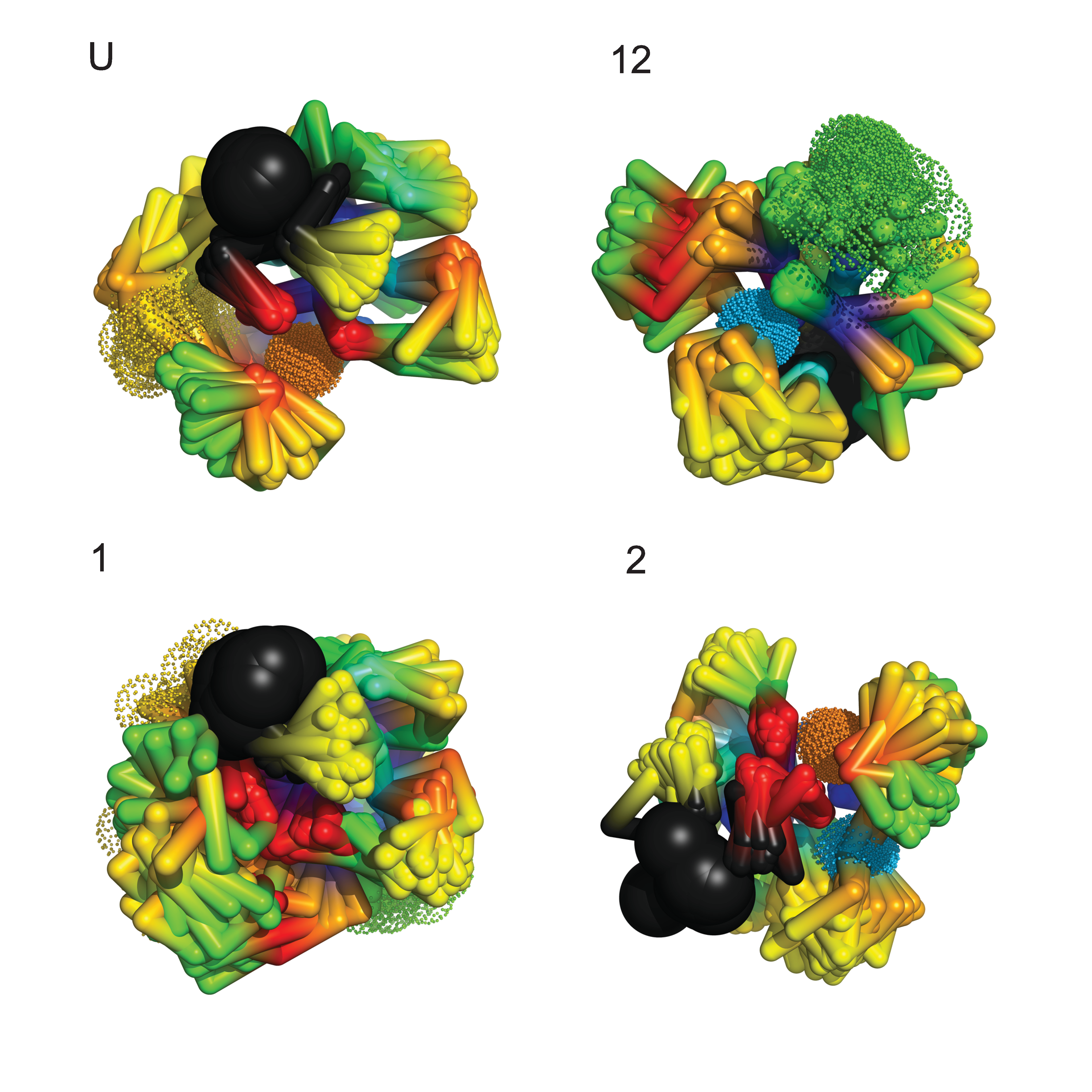

Supplement: S4 Fig — Panel (U) corresponds to the initial, un–mutated sequence. Panels (1) and (2) correspond to the single mutants R1 (orange) → G1 (green) and T12 (yellow) → I12 (light blue), respectively. Panel (12) corresponds to the double mutant. Dotted spheres indicate the positions of mutated amino acids. Each ensemble ΔΓ⋆ is aligned to its corresponding reference fold, x⋆, using methods described in ref. [14] For clarity, each figure panel includes the 30 closest structures to x⋆. Ensembles are rotated to reveal the positions of mutated monomers. (TIFF) [file pone.0166739.s004.tiff]

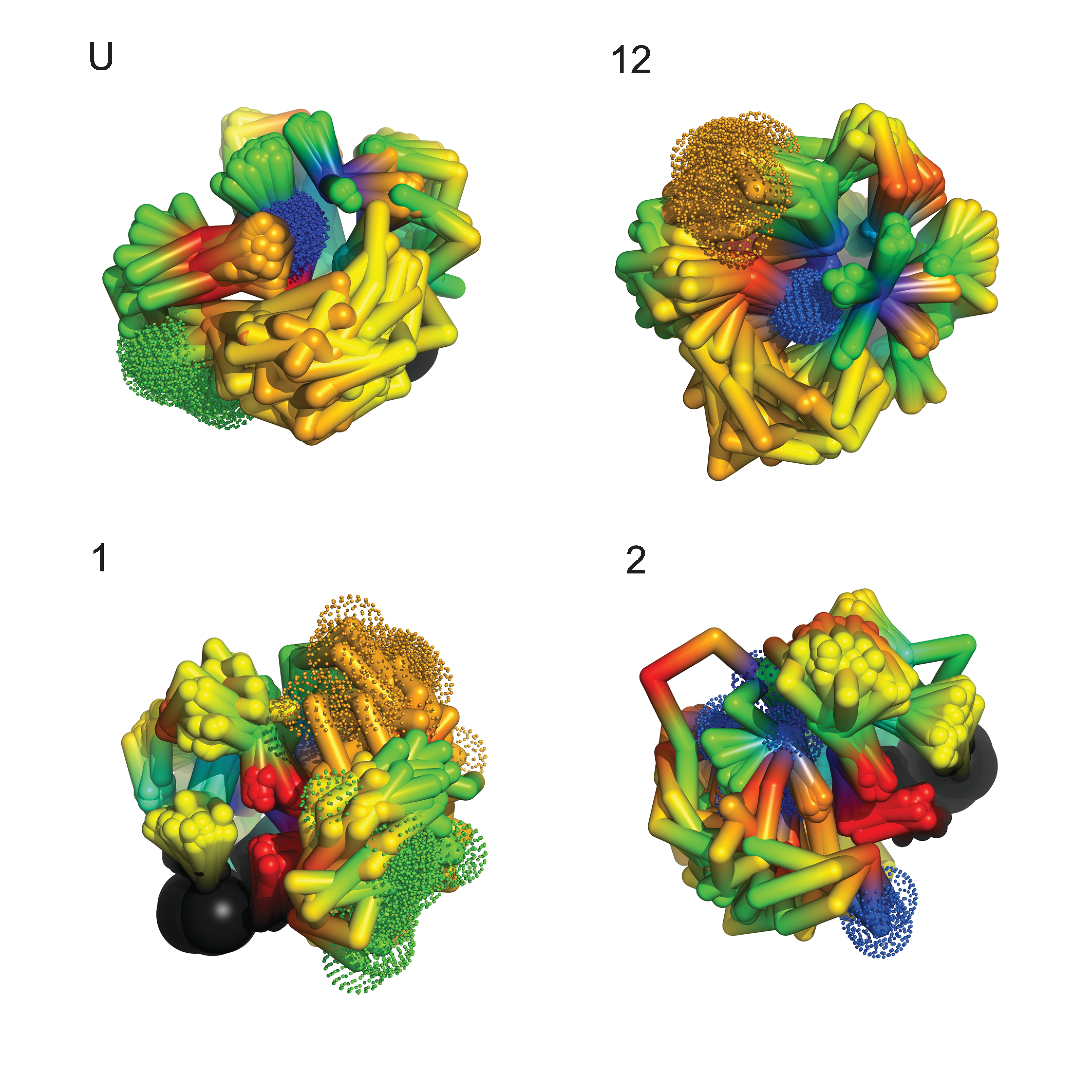

Supplement: S5 Fig — Panel (U) corresponds to the initial, un–mutated sequence. Panels (1) and (2) correspond to the single mutants W4 (blue) → S4 (yellow) and G10 (green) → V10 (blue), respectively. Panel (12) corresponds to the double mutant. Ensembles are arranged as described in S5 Fig. (TIFF) [file pone.0166739.s005.tiff]

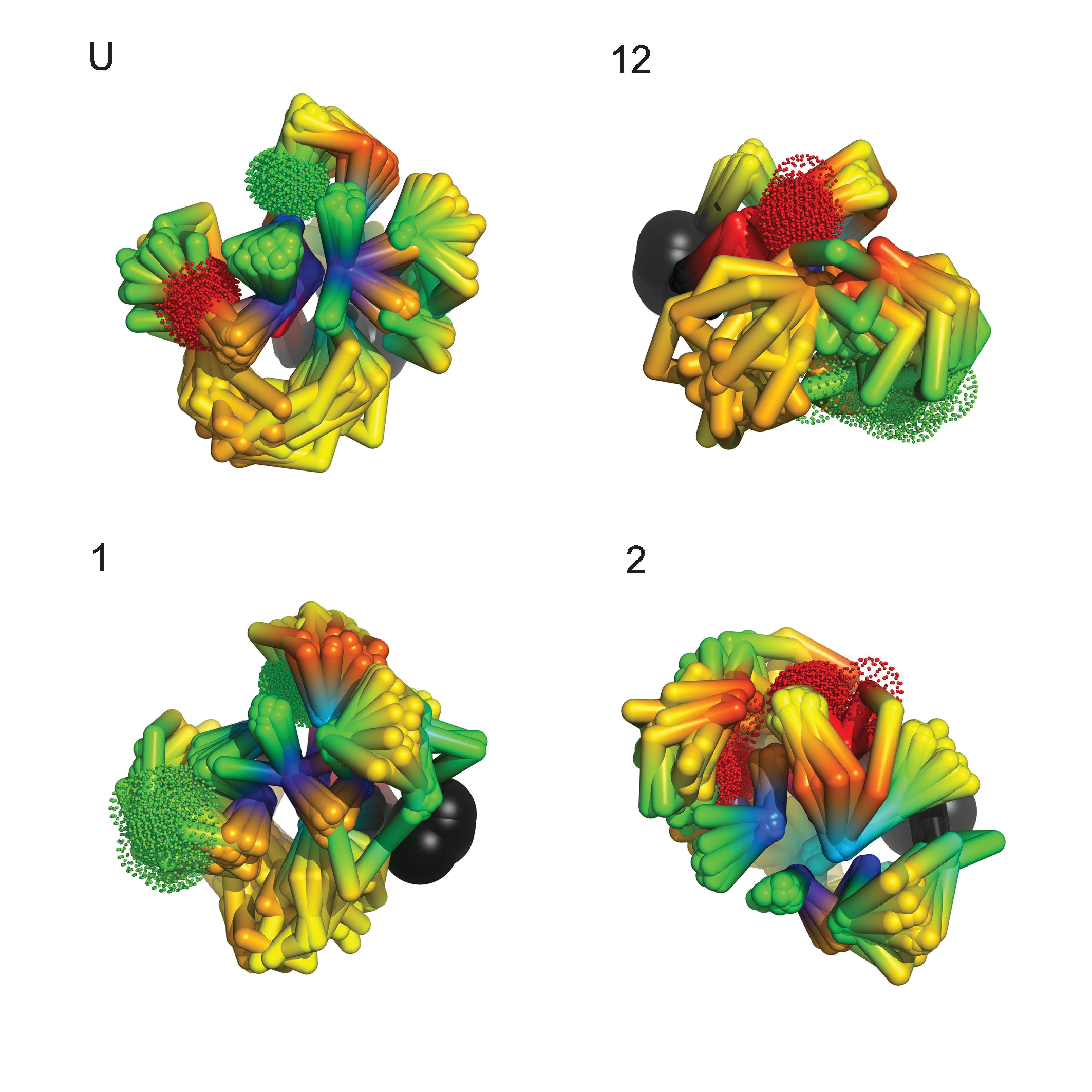

Supplement: S6 Fig — Panel (U) corresponds to the initial, un–mutated sequence. Panels (1) and (2) correspond to the single mutants E6 (red) → G6 (green) and A23 (green) → E23 (red), respectively. Panel (12) corresponds to the double mutant. Ensembles are arranged as described in S5 Fig. (TIFF) [file pone.0166739.s006.tiff]

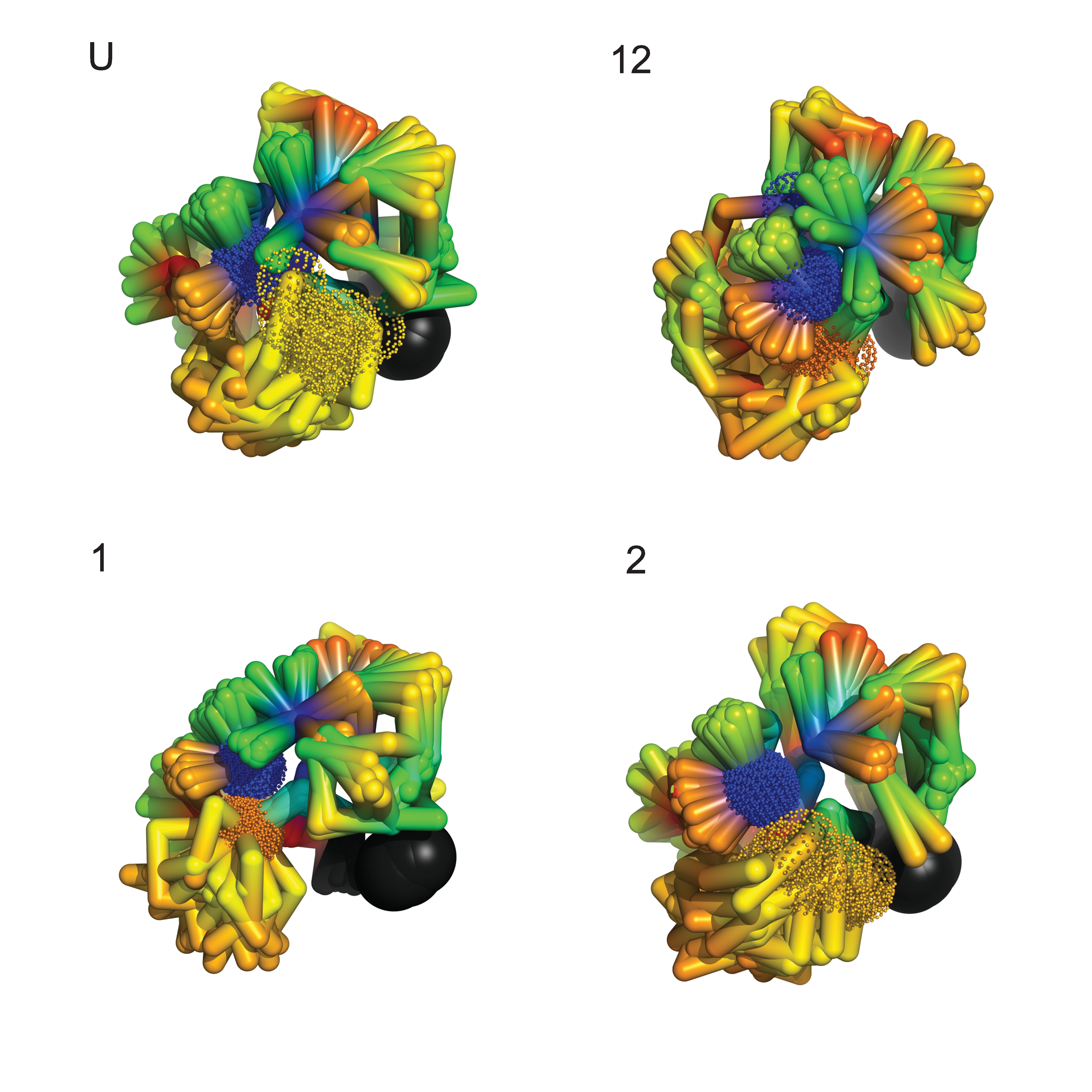

Supplement: S7 Fig — Panel (U) corresponds to the initial, un–mutated sequence. Panels (1) and (2) correspond to the single mutants T15 (yellow) → R15 (orange) and W4 (blue) → C4 (blue), respectively. Panel (12) corresponds to the double mutant. Ensembles are arranged as described in S5 Fig. (TIFF) [file pone.0166739.s007.tiff]

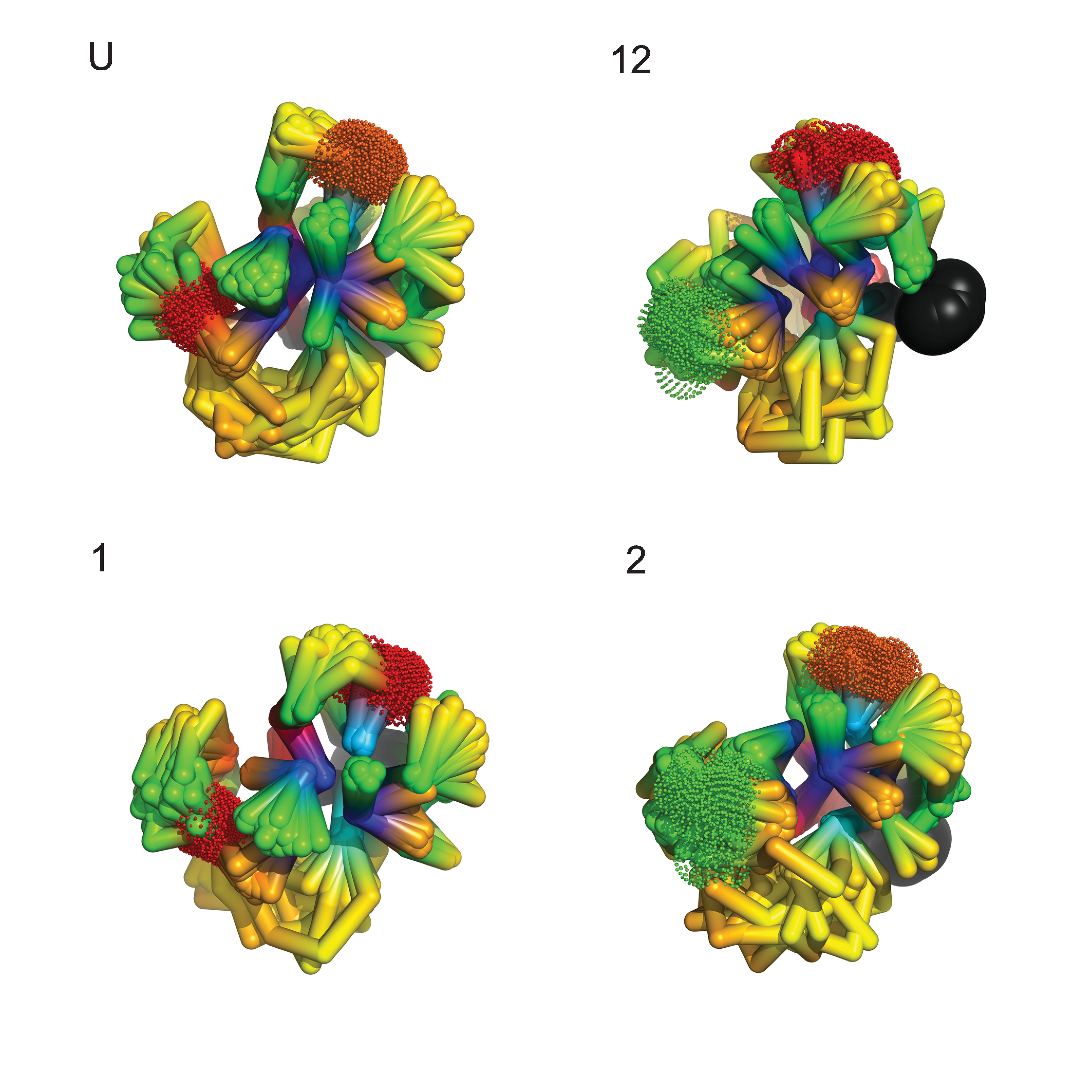

Supplement: S8 Fig — Panel (U) corresponds to the initial, un–mutated sequence. Panels (1) and (2) correspond to the single mutants Q25 (orange) → E25 (red) and E6 (red) → G6 (green), respectively. Panel (12) corresponds to the double mutant. Ensembles are arranged as described in S5 Fig. (TIFF) [file pone.0166739.s008.tiff]

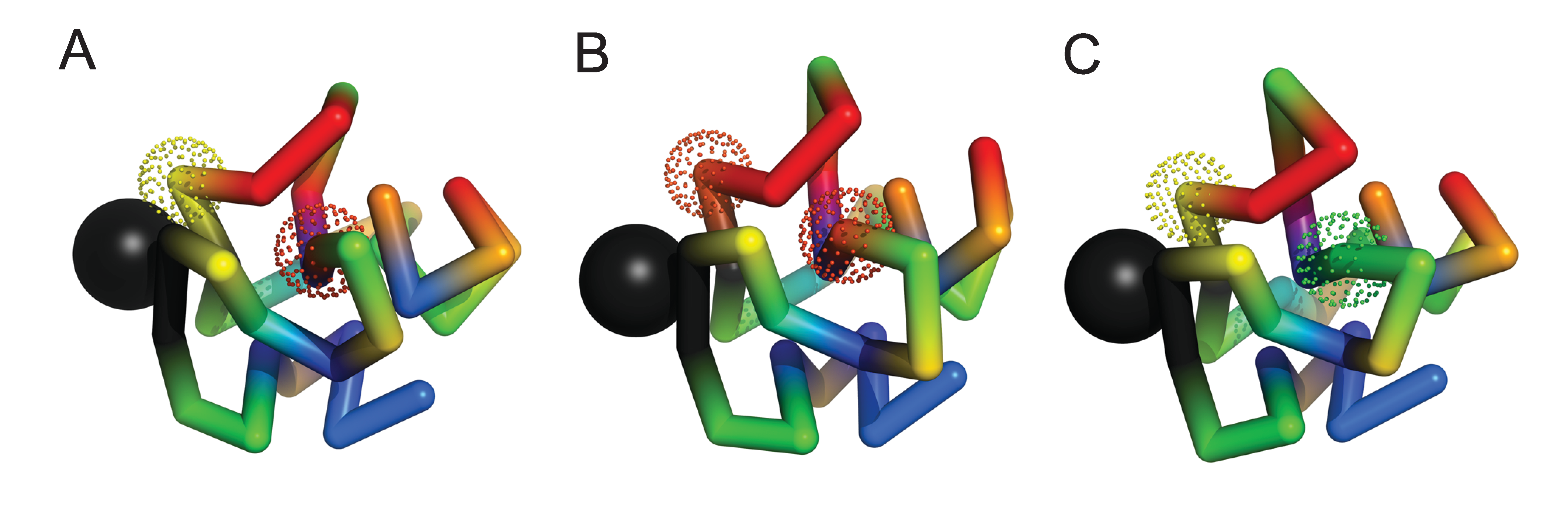

Supplement: S9 Fig — Individual mutations N13 (yellow) → D13 (red) and D19 (red) → Y19 (blue–green) in panels (B) and (C) are nearly neutral, with ΔP1≃−0.07 and ΔP2≃−0.1 respectively (ΔP12≃−0.43). Both N13 and D19 are ordered in the initial ensemble, and both D13 and Y19 are ordered in the single mutant ensembles. Single mutations do not significantly alter the reference structure of the initial sequence. In the double mutant, the mutation, D19 → Y19, reduces the specificity of interactions with its neighbors, allowing for greater conformational freedom of the binding site, making it more susceptible to the negative effect of the mutation, N13 → D13; The mutated amino acids Y19 and D13 form favorable contacts during folding, which disrupts the binding site in the quenched ensemble. The distance between mutated positions in panel (A) is R≃7.3 Angstroms. (TIFF) [file pone.0166739.s009.tiff]
